# Redox regulation, thioredoxins, and glutaredoxins in retrograde signalling and gene transcription

**DOI:** 10.1093/jxb/erad270

**Published:** 2023-07-15

**Authors:** Francisca Sevilla, Maria Carmen Martí, Sabrina De Brasi-Velasco, Ana Jiménez

**Affiliations:** Abiotic Stress, Production and Quality Laboratory, Department of Stress Biology and Plant Pathology, CEBAS-CSIC, Murcia, Spain; Abiotic Stress, Production and Quality Laboratory, Department of Stress Biology and Plant Pathology, CEBAS-CSIC, Murcia, Spain; Abiotic Stress, Production and Quality Laboratory, Department of Stress Biology and Plant Pathology, CEBAS-CSIC, Murcia, Spain; Abiotic Stress, Production and Quality Laboratory, Department of Stress Biology and Plant Pathology, CEBAS-CSIC, Murcia, Spain; Universidad de Sevilla, Spain

**Keywords:** Gene expression, glutaredoxin, plant stress, redox regulation, retrograde signalling, ROS, thioredoxin

## Abstract

Integration of reactive oxygen species (ROS)-mediated signal transduction pathways via redox sensors and the thiol-dependent signalling network is of increasing interest in cell biology for their implications in plant growth and productivity. Redox regulation is an important point of control in protein structure, interactions, cellular location, and function, with thioredoxins (TRXs) and glutaredoxins (GRXs) being key players in the maintenance of cellular redox homeostasis. The crosstalk between second messengers, ROS, thiol redox signalling, and redox homeostasis-related genes controls almost every aspect of plant development and stress response. We review the emerging roles of TRXs and GRXs in redox-regulated processes interacting with other cell signalling systems such as organellar retrograde communication and gene expression, especially in plants during their development and under stressful environments. This approach will cast light on the specific role of these proteins as redox signalling components, and their importance in different developmental processes during abiotic stress.

## Teaser

Redox regulation of retrograde signalling and gene expression plays a role in abiotic stress adaptation and development.

## Introduction

Plants are subjected to a wide variety of stressful biotic and abiotic conditions that trigger signal transduction pathways, provoking molecular, metabolic, and physiological responses to regulate their adaptation and survival. This is particularly alarming nowadays when climate change is limiting plant productivity and yield in a scenario of a growing population (Choudhury *et al*., 2017; Lamers *et al*., 2020). Understanding how plants respond to this changing environment is an important issue for improving plant tolerance.

**Fig. 1.**
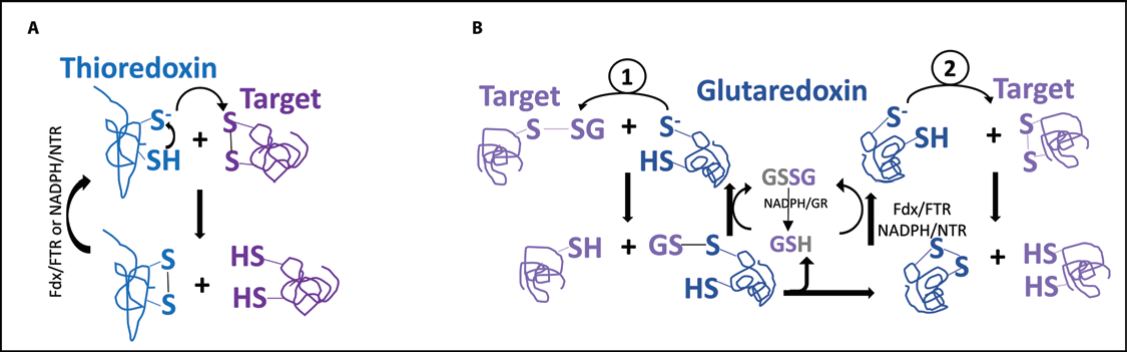
Schematic representation of TRX and GRX catalytic mechanisms. (A) Thioredoxin (TRX)-dependent protein disulfide reduction: the thiolate form of a Cys residue in the active centre of TRX attacks a disulfide bond of a specific target protein and the transient complex is resolved by a second Cys, resulting in the target being reduced, while TRX is oxidized. Regeneration of the reduced TRX is carried out by the ferredoxin (Fd)-dependent ferredoxin reductase (FTR) or NADPH-dependent thioredoxin reductase (NTR). (B) Glutaredoxin (GRX) uses GSH for the reduction of (1) glutathione disulfide substrates through a monothiol mechanism (left side) with a thiolate attack on the S–SG form of a specific target, or (2) non-glutathione disulfide substrates through a dithiol mechanism (right side) with a thiolate attack on the S–S form of a specific target. GSH is regenerated by NADPH-dependent glutathione reductase (GR), while reduced GRX is regenerated by FTR or NTR.

Growing evidence points to signalling molecules, such as reactive oxygen species (ROS), as part of the signal transduction pathways induced by stressful conditions. H_2_O_2_ is the most relevant ROS due to its reactivity, diffusivity, and prolonged half-life compared with others such as the radicals superoxide (O_2_·^–^) and hydroxyl (·OH) or the non-radical species singlet oxygen (^1^O_2_). Biotic and abiotic stresses are characterized by associated oxidative stress generated by an increase in ROS in the different cell compartments. Cysteine (Cys) is one of the main amino acid targets of ROS, and Cys thiols (-SH) are weak acids in equilibrium with the deprotonated thiolate form (-S^−^) in the physiological range of pH. For its part, -S^–^ is more sensitive to the intracellular redox environment than -SH and is susceptible to oxidative modifications, functioning as a redox switch (Trost *et al*., 2017). During redox signalling, H_2_O_2_ oxidizes the thiolate anion to the sulfenic form (Cys-SOH) which can react with another thiolate to form an intra- or intermolecular disulfide bond (S–S). Higher levels of oxidation oxidize the thiolate to a sulfinic (-SO_2_H) or sulfonic (-SO_3_H) species, the latter being irreversible (reviewed by Sevilla *et al*., 2015). Interestingly, the reversion of some of these oxidized forms can serve as a signal transduction mechanism to ensure transient signalling and an adequate response to a stress situation, while also preventing irreversible overoxidation of the proteins, which usually negatively affects their function (Mukherjee, 2021). Therefore, ROS must be tightly controlled by the antioxidant system in collaboration with the redox system composed of proteins able to transfer electrons from the input elements to downstream target proteins. These transmitters are a large family of oxidoreductase proteins in plants, including the so-called ‘redoxins’: thioredoxins (TRXs), TRX-like proteins, and glutaredoxins (GRXs), with 44 genes encoding TRXs or TRX-like proteins and 27 genes encoding GRXs in *Arabidopsis thaliana* (Couturier *et al*., 2009; Meyer *et al*., 2012). TRXs are ancient ubiquitous enzymes present in prokaryotic and eukaryotic organisms, able to catalyse reversible disulfide bond formation in specific target proteins in the different cell compartments, in this way regulating their structure and function (reviewed by Calderón *et al*., 2018; Martí *et al*., 2020; Trnka *et al*., 2020) (Fig. 1). In *A. thaliana*, there are 21 genes of typical TRXs in family I, with TRXs *m*1-4, *f*1-2, *x*, *y*1-2, *s*, and *z* located in the plastids, eight TRXs *h* in the cytosol, nucleus, plasma membrane, and endoplasmic reticulum (ER), and TRX*o*1-2 located in the mitochondria and nucleus (reviewed by Calderón *et al*., 2018). Family II includes fusion proteins with one or more TRX domains coupled to additional domains, such as NADPH-dependent thioredoxin reductase C (NTRC), which contains an N-terminal NADPH-dependent thioredoxin reductase (NTR) and a C-terminal TRX domain (Pérez-Ruiz *et al*., 2006; Martí *et al*., 2020). GRXs are small disulfide oxidoreductases that catalyse the reversible reduction of disulfide bridges and glutathione (GSH)-mixed disulfides (deglutathionylation) through a dithiol or monothiol mechanism, respectively, using GSH as electron donor (Rouhier *et al*., 2015; Begas *et al*., 2017). GSH is the general reductant of oxidized GRXs, although TRXs are also able to act in the same way (reviewed by Zaffagnini *et al*., 2019) (Fig. 1). There are five GRX subgroups based on similarity and characteristics of the active site, of which III and IV are specific to vascular plants (reviewed by Meyer *et al*., 2012). In *A. thaliana*, there are six genes in group I (C[P/G/S]Y[C/S] in the catalytic site), with members present in the cytosol and plastids, four proteins in group II (CGFS) in the cytosol, plastid, nucleus, and mitochondria, and 21 members in group III (CCx[C/S/G] now named ROXY GRXs) with cytoplasmic and nuclear members. Group IV contains poorly characterized proteins with a GRX domain followed in the C-terminus by four CxxC repeats (Navrot *et al*., 2006), while group V (CPF[C/S]) has six members distributed among the cytosol, mitochondria, and chloroplasts. TRXs and GRXs function as oxidoreductases regulating the oxidative state of Cys residues of target proteins, and therefore their activity, localization, and structure.

Retrograde signalling from the different cell compartments to the nucleus is a process that allows plants to cope with stressful situations arising from endogenous or external stimuli that alter the functioning of organelles. The process includes the regulation of nuclear gene expression in order to establish a coordinated transcription with the organellar genomes. This process involves several signalling players, from sensors to transmitters and transductors, and allows the fine-tuning of organelle biogenesis (biogenetic control) and response to stress (operational control) (Crawford *et al*., 2018; Wang *et al*., 2020). In this review, we focus on post-translational modifications (PTMs) of Cys that have been associated with ROS-mediated organellar retrograde signalling and with ROS modulation of transcription factors (TFs) responsible for changes in gene expression, which might or might not be directly related to organelle communication. In both processes, we pay special attention to the role of TRXs and GRXs, in an attempt to elucidate their importance in the adaptation of plants to the increasingly unfavourable growth conditions they face.

## Redox regulation of retrograde signalling and participation of TRX and GRX

Chloroplasts and mitochondria are organelles that have essential functions in plant energy conversion and which play a central role in a variety of primary and secondary metabolic pathways. Both organelles participate in retrograde signalling, although the mechanism they use to integrate the different signals is unknown (Wang *et al*., 2020). Moreover, other cellular compartments may participate in retrograde signalling, and integrate the signals and responses provoked by a specific stress signal, allowing the plant’s adaptation. In this section, we summarize evidence of the involvement of redox regulation in the different cell compartments involved in retrograde signalling events at three levels: retrograde signals generated, transducers that relay the signals to the nucleus, and TFs involved in the process that regulate nuclear gene expression.

### Chloroplasts in retrograde signalling

**Fig. 2.**
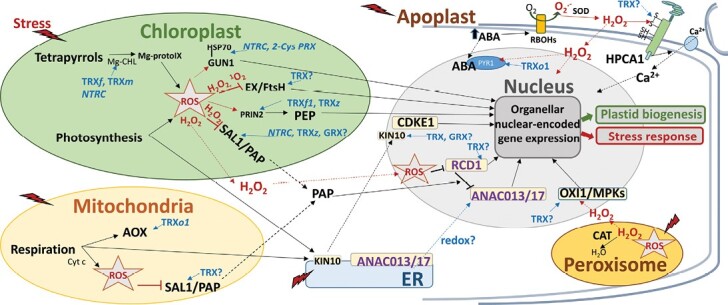
TRX/GRX-dependent redox regulation of retrograde signalling during organelle biogenesis and stress response. Retrograde signalling from organelles and cell compartments in which the components regulated by TRX or GRX systems are presented, allowing a nuclear response through the activity of transcription factors (violet letters) or kinases (black letters). The participation of redox regulation, TRXs or GRXs (? denotes hypothesized) together with the involvement of ROS is derived from evidence reported in the literature, as described in the text. Dashed lines represent movement of the protein/metabolite and arrows represent an effect on the protein/compound. ABA, abscisic acid; ANAC; no apical meristem/Arabidopsis transcription activation factors/cup shaped cotyledon; AOX, alternative oxidase; CAT, catalase; CDKE1, cyclin-dependent kinase E1; 2-Cys PRX, 2 cysteine peroxiredoxin; Cyt c, cytochrome *c* pathway; ER, endoplasmic reticulum; EX, executer; FtsH, filamentation temperature-sensitive ATP-dependent zinc metalloprotease; GRX, glutaredoxin; GUN1, genome uncoupled 1; HPCA1, hydrogen-peroxide-induced Ca^2+^ increases 1; HSP70, heat shock protein 70; KIN10, SnRK1-Sucrose non-fermenting Related protein Kinase 1; Mg-CHL, Mg-chelatase chlorophyll subunit; Mg-protoIX, Mg-protoporphyrin IX; MPK, mitogen-activated protein kinase; NTRC, NADPH-dependent thioredoxin reductase C; PAP, 3ʹ-phosphoadenosine 5ʹ-phosphatase; PEP, plastid-encoded RNA polymerase; PRIN2, Plastid Redox INsensitive2; PYR1, pyrabactin resistance1; RBOHs, NADPH oxidase/respiratory burst oxidase homologues; RCD1, Radical-Induced Cell Death; OXI1, Oxidative signal-inducible1; ROS, reactive oxygen species; SAL1, 3ʹ(2ʹ), 5ʹ-bisphosphate nucleotidase; SOD, superoxide dismutase; TRX, thioredoxin.

Chloroplasts produce several ROS during photosynthesis that have been proposed to activate or participate in retrograde signalling (Fig. 2). Singlet oxygen (^1^O_2_) generated by the excitation at PSII may provoke an irreversible photoinhibition and/or oxidation of lipids, carotenoids, and proteins (Dogra and Kim, 2020). ^1^O_2_ is also known as a key regulator of the retrograde signalling associated with the induction of antioxidant defences such as thylakoid ascorbate peroxidase (tAPX) in *A. thaliana* (Laloi *et al*., 2007) and with localized programmed cell death, through the participation of, among others, key players such as EXECUTER (EX) proteins and phytohormones (reviewed by Laloi and Havaux, 2015). These triggered events finally collaborate with other ROS involved in retrograde signalling such as O_2_·^–^ and H_2_O_2_ to increase resistance to different stress conditions (Uberegui *et al*., 2015; Carmody *et al*., 2016) as we shall describe below. Another source of ^1^O_2_ are tetrapyrroles, the intermediates of haem and chlorophyll (Chl) biosynthesis, considered as key playes in chloroplast retrograde signalling because they act as strong photosensitizers (von Gromoff *et al*., 2008). The analysis of *A. thaliana* mutants in ^1^O_2_ metabolism such as the *flu* mutant has revealed some clues regarding retrograde signalling in chloroplasts. *flu* is a negative regulator of tetrapyrrole biosynthesis so it accumulates protochlorophyllide (Pchlide), a potent photosensitizer that is an ^1^O_2_ light-dependent generator (Meskauskiene *et al*., 2001). This mutant has pointed to a role for the nuclear-encoded chloroplast proteins EX1 and EX2, associated with the thylakoid membrane, as transducers in the ^1^O_2_-triggered chloroplast-to-nucleus retrograde signalling pathways under acclimation to hight light stress (reviewed by Mielecki *et al*., 2020). Oxidation of Trp643 in EX1 by ^1^O_2_ induces a conformational change and protein degradation by the plastid ATP-dependent zinc metalloprotease (FtsH), affecting the repair of PSII (Carmody *et al.*, 2016). In photosynthetic organisms, FtsH forms heterohexamers and, recently, a new method of redox regulation of oligomerization has been demonstrated in *Chlamydomonas reinhardtii*, the proteolytic activity of this protease also being redox regulated, both *in vitro* and *in vivo* (Wang *et al*., 2017). Moreover, these authors suggested that reduction of the disufide bridges formed in the oligomers favouring the proteolytic activity may be controlled by TRX. Another association between ^1^O_2_ and oxidative stress, in this case mediated by H_2_O_2_, is the observation that overexpression of tAPX in the *flu* mutant provokes a strong decrease of transcript levels related to the EX1 pathway (Laloi *et al*., 2007). Another example in which tetrapyrrole metabolism is involved in retrograde signalling is via ^1^O_2_ generation mediated by Mg-protoporphyrin IX (Mg-protoIX), an intermediate in Chl biosynthesis (reviewed by Whittmann *et al*., 2021). Because the TRX system has been reported as a redox regulator of several enzymes involved in this biosynthesis pathway, redox regulation may affect retrograde signalling through control of tetrapyrrole metabolism. Signals from Mg-protoIX are considered to be associated with inhibition of photosynthesis and the generation of responses against stress (Zhang *et al.*, 2016). The incorporation of Mg into to protoIX is catalysed by Mg-chelatase, a heterotrimeric enzyme composed of subunits CHLI, CHLD, and CHLH (CHLorophyll I, D, and H) (Luo *et al*., 2012). Redox regulation of CHLI activity was described as being carried out by TRX *f*, *m*, and NTRC (Pérez-Ruiz *et al.*, 2014). NTRC also regulated CHLH and other upstream enzymes such as glutamyl-transfer RNA reductase1 (GluTR1; Richter *et al*., 2013, 2018), implying the relevance of NTRC in Chl biosynthesis at different levels of the pathway (reviewed by Wittmann *et al*., 2021). The accumulation of Mg-protoIX triggers plastid-to-nucleus signalling mediated by the TF ABA INSENSITIVE4 (ABI4), as reported in Genome UNcoupled (*gun*) retrograde signalling mutants. Six of these mutants have been selected in *A. thaliana* because the expression of the PhANG *LHCB* (light-harvesting chlorophyll *a*/*b*-binding protein) is uncoupled from the functional state of the chloroplasts (Koussevitzky *et al*., 2007), although, more recently, the involvement of ABI4 in biogenic chloroplast retrograde communication has been questioned after detailed analysis of *Atabi4* mutants (Kacprzak *et al*., 2019). ABI4 has also been linked to the regulation of *AOX1a* expression in response to mitochondrial signals. In fact, the application of abscisic acid (ABA) induces the expression of *AOX1a*, which means that ABI4 can act as a positive and negative regulator, or that other ABA-responsive factors may be involved (Giraud *et al*., 2009; Wang *et al*., 2020). Thus, *AOX1a* expression is sensitive to perturbations in the redox/energy status of both plastids and mitochondria. In chloroplasts, GUN1 is a pentatricopeptide repeat protein that takes part in multiple processes to coordinate nuclear gene expression in response to plastid signals (Fortunato *et al*., 2022). In response to GUN1-derived signals, ABI4 TF represses PhANGs by preventing DNA binding of factors needed for their expression. Using lincomycin (a repressor of PhANGs), GUN1 has been shown to mediate an H_2_O_2_-dependent oxidized environment which may represent a redox signal, providing valuable insight into the role of chloroplast ROS and redox changes in biogenic retrograde communication (Karpinska *et al*., 2017). Under optimal physiological conditions during plastid biogenesis, GUN1 also indirectly influences O_2_·^–^ accumulation through the regulation of superoxide dismutase (SOD) and APX enzyme activities, playing a role in protecting the organelles from potential oxidative damage (Fortunato *et al*., 2022). In this context, redoxins are important players in the redox regulation of key sensors. Together with 2-Cys peroxiredoxin (2-Cys PRX), NTRC participate in retrograde signalling through interaction with the complex GUN1–cpHSP70 (chloroplast heat shock protein 70). cpHSP70 is a chaperone involved in importing chloroplast proteins to the organelle during biogenesis (Cejudo *et al*., 2021). The activation of plastid-encoded RNA polymerase (PEP) during chloroplast development has been described as another retrograde signal that promotes PhANG expression (Kindgren *et al*., 2012; Wang *et al*., 2023). Plastid Redox INsensitive2 (PRIN2), a redox-regulated protein required for full PEP-driven transcription, is a target of TRX*f*1 and TRX*z*, which are responsible for the *in vitro* reduction of S–S bridges of PRIN2 dimers in the generation of active monomers. This mechanism provides a redox TRX-mediated retrograde process that links photosynthetic electron transport to activation of PhANGs (Díaz *et al.*, 2018).

The role of GRX in chloroplast retrograde signalling has been less explored, although a meta-analysis of this process in *A. thaliana* revealed GRX as one of the components of the core module of genes responding to six different treatments provoking retrograde signalling (Glässer *et al*., 2014). Similar to the redox regulation of TRX in chlorophyll metabolism, some evidence of a possible role for GRXs in Chl synthesis has been reported: for example, proteomic studies in *C. reinhardtii* and *Synechocystis* sp. PCC6803 have revealed that several enzymes of this pathway are targets of glutathionylation, a PTM specifically reversed by GRXs (Zaffagnini *et al*., 2012), while plastidial class II GRXS14 participates in the maturation of Fe–S proteins that are incorporated in numerous enzymes of the Chl biosynthetic pathway. Moreover, GRXS14 deficiency in *A. thaliana* resulted in a drastic decrease in the Chl content in dark conditions, while overexpression was associated with lower levels of proteins of the Fe–S machinery, in this way regulating Chl metabolism (Rey *et al*., 2007). Thus, the modulation of Chl metabolism by GRX may have an effect on retrograde signalling events derived from components of the biosynthetic pathway, although this aspect needs further investigation.

In addition to Trp, ^1^O_2_ may provoke oxidation in proteins via histidine, tyrosine, or methionine, and more slowly via cysteine and cystine (Davies, 2003). The reaction with thiols induces the oxidation of sulfur and sulfonic acids (Dmitrieva *et al*., 2020), and TRXs and GRXs may play a role in the regulation of the oxidative state of these ^1^O_2_-oxidized proteins. A new mechanism has been described by means of which ^1^O_2_ oxidizes disulfide bridges in proteins, forming a thiosulfinate that may react with GSH (or other thiols), generating glutathionylated proteins (Jiang *et al*., 2021). It would be interesting to know whether GRX could be involved in the modulation of this glutathionylation. Related to this, a role for TRX*y* and TRX*h*1 in ^1^O_2_ stress has been suggested through the redox modulation of GPX5, one of the main enzymes involved in the defence against ^1^O_2_ in *C. reinhardtii* (Ledford *et al*., 2007; Fischer *et al*., 2009).

Tocopherol, β-carotene, and plastoquinone are efficient quenchers of ^1^O_2_ and, interestingly, human TRX has been demonstrated as a very efficient ^1^O_2_ quencher, the catalytic and the structural cysteines being required for its ROS-scavenging properties (Das and Das, 2000). If ^1^O_2_ is not quenched, it can trigger the up-regulation of genes involved in defence against photo-oxidative stress (Krieger-Liszkay *et al*., 2008). The quenching of ^1^O_2_ by β-carotene generates β-cyclocitral (β-CC), which might act as a mobile signal to enhance downstream pathways (Dogra and Kim, 2020), reprogramming gene expression in response to stress, as reported under high light (Mullineaux *et al*., 2018). The β-CC- and EX1-dependent pathways have only a small number of common ^1^O_2_-responsive genes, β-CC-mediated signalling being independent of EX1/EX2 (Shumbe *et al*., 2016). Interestingly, genes regulated by β-CC include some that are involved in xenobiotic detoxification mechanisms, a process with which RX480/ROXY19 has been related through its competition for binding with AtTGA2 TFs, in this way inhibiting the detoxification response induced by β-CC (D’Alessandro *et al*., 2018).

In chloroplasts, another major source of ROS is the photoreduction of O_2_ at PSI (Mehler reaction) that produces superoxide anion (O_2_·^–^), which generates H_2_O_2_ as a result of thylakoid-bound and stromal SODs. H_2_O_2_ can oxidize the Calvin–Benson cycle enzymes that are regulated by the TRX system, mainly by Trx*f* (reviewed by Michelet *et al*., 2013). H_2_O_2_ is closely controlled by the ascorbate–glutathione (ASC–GSH) cycle components and by thiol-dependent peroxidases (TPXs) including 2-Cys PRX and glutathione peroxidase-like (GPX) enzymes (Barranco-Medina *et al*., 2008; Cejudo *et al*., 2021; Foyer and Hanke, 2022). Chloroplast H_2_O_2_ also plays an important role in retrograde signalling by inhibiting some key regulators of chloroplasts retrograde signalling such as 3ʹ(2ʹ),5ʹ-bisphosphate nucleotidase (SAL1) which is thought to act as a sensor of oxidative stress in these organelles. H_2_O_2_ provokes a conformational change oxidizing Cys thiols of SAL1 as a response to redox changes in chloroplast GSH (Chan *et al*., 2016). AtSAL1 phosphatase activity is suppressed by dimerization, intramolecular disulfide formation, and glutathionylation, resulting in the accumulation of its substrate, 3ʹ-phosphoadenosine 5ʹ-phosphatase (PAP) (Chan *et al*., 2016). As a consequence of this oxidative inactivation, PAP is translocated from the chloroplasts to the nucleus and acts as retrograde signal to regulate the plastid redox-associated nuclear genes (PRANGs) involved in responses to high light, drought, and programmed cell death (Estavillo *et al*., 2011; Bruggeman *et al*., 2016). In fact, PAP-accumulating *sal1* mutants are highly tolerant to drought stress (Estavillo *et al*., 2011). In this context, the TRX system may play a key role due to the observation that perturbation in chloroplast redox homeostasis, as occurs in *ntrc* mutants, influences SAL1 activity by regulating the expression of PRANGs, reflecting the influence of redox modulation on AtSAL1 activity and PAP accumulation (Chan *et al*., 2016). Also, TRX*z* has been described as being involved in the activation of PAP through two interaction partner fructokinase-like proteins during chloroplast development and early seedling development (Arsova *et al*., 2010). In this scenario, GRX may have a role in the recovery of the SAL1 activity inhibited by deglutathionylation, a mechanism that needs to be further studied. Interestingly, SAL1 is targeted to chloroplasts and mitochondria, which suggests that the PAP-mediated chloroplast retrograde signalling is related to mitochondrial signalling (Estavillo *et al*., 2011). Additionally, recent evidence suggests that the conservation and coordination of the SAL1–PAP pathway and ABA is linked to the regulation of stomatal closure and adaptation to a variety of terrestrial habitats during the diversification of land plants (Pornsiriwong *et al*., 2017; Zhao *et al*., 2019).

Due to its relatively high stability, H_2_O_2_ could be a mobile signal acting as a transducer and initiator of retrograde signalling from chloroplasts. The transit of H_2_O_2_ from chloroplasts to nuclei may occur through passive diffusion or membrane channels formed by aquaporins, although their function in H_2_O_2_ transport requires further confirmation. A direct H_2_O_2_ transfer from chloroplast to nuclei following H_2_O_2_ application has been reported to occur through tube-like structures termed stromules that connect the chloroplast to nuclei during innate immunity (Caplan *et al*., 2015), which may be involved in retrograde signalling. Also, the transfer of H_2_O_2_ from surrounding chloroplasts to the nucleus has been described as a response to high light (Expósito-Rodríguez *et al*., 2017). In this context, NTRC is a key regulator of the chloroplast redox state and controls the formation of stromules in response to high light signals, with the down-regulation of NTRC generating an increase in stromules (Brunkard *et al*., 2015).

### Mitochondria in retrograde signalling

Like chloroplasts, mitochondria can signal to the nucleus their functional state to guide the expression of responsive genes via MRR (mitochondrial retrograde regulation) through different emitted signals (Pfannschmidt *et al*., 2020). However unlike chloroplasts, the mechanism by which these signals are transduced into the nucleus is less well understood, despite the fact that some downstream components have been described, mainly using alternative oxidase (AOX) as a key marker (Fig. 2).

The mitochondrial electron transport chain (mtETC) produces O_2_·^–^ as a by-product under normal and stress conditions, which is scavenged by Mn-SOD, producing H_2_O_2_ (Sevilla *et al*., 1982). H_2_O_2_ is further reduced in the ASC–GSH cycle or by other peroxidase systems such as GPXLs and PRXs (Jiménez *et al*., 1997; Barranco-Medina *et al*., 2008; Foyer and Noctor, 2011; Iglesias-Baena *et al*., 2011), which are regenerated by the TRX/NTR system, ultimately using NADPH as a reductant (Banze and Follmann, 2000; Laloi *et al*., 2001; Gelhaye *et al*., 2005; Martí *et al*., 2009). Mitochondrial GRXs play a main role in Fe–S cluster protein biosynthesis (Couturier *et al.*, 2015). H_2_O_2_ may either enter or leave the mitochondria through voltage-dependent anion channels or aquaporins, which may have a signalling role (Smirnoff and Arnaud, 2019), although their participation in MRR has not been fully confirmed.

AOX is a component of the respiratory chain that diverts electrons to O_2_ with the production of H_2_O, encouraging dissipation of the proton gradient. AOX is considered as a key marker for monitoring the activation of mitochondrial retrograde signalling (MRS) following mitochondrial dysfunction, often associated with ROS production, Mn-SOD activity, and cellular oxidative stress (Van Aken, 2021). Some regulators of MRS which modulate *AOX* expression have been identified by forward genetic screening in *A. thaliana*, and, among them, the NAC TFs ANAC017 and ANAC013 mediate a ROS-related retrograde signal originating from mitochondrial complex III (De Clercq *et al*., 2013). Both proteins are bound to the ER membrane and are released and translocated to the nucleus by a RHOMBOID-LIKE 2 protease (Eysholdt-Derzsó *et al*., 2023). In the nucleus, ANAC017 and ANAC013 activate the mitochondrial dysfunction stimulon (MDS) genes including *AOX* genes, sulfotransferase12 (*SOT12*), and *ANAC013* itself (Van Aken *et al*., 2016). Additionally, mitochondrial respiration can alleviate thiol-based reductive stress in the ER. The ER is in charge of the processing of proteins for secretion, including oxidative protein folding which requires the oxidation of Cys residues. Recently, it has been shown that reductive stress triggers ANAC017-mediated retrograde signalling to safeguard the ER by boosting mitochondrial respiratory capacity (Fuchs *et al*., 2022). Despite the identification of ANAC017 and ANAC013, there are still gaps in our knowledge of the upstream mechanism by which the ANAC proteins respond to ROS and are cleaved from the ER; neither is it known whether their release from the ER is redox regulated (Fig. 2).

The identification of ANAC017 as a TF acting in MRS has allowed the recognition of connected pathways between the retrograde signals of both mitochondria and chloroplasts. In this sense, Shapiguzov *et al*. (2019) showed that the nuclear Radical-Induced Cell Death1 protein (RCD1) acts as a connecting link in the ROS-dependent signalling pathways from chloroplasts and mitochondria. RCD1 interacts with ANAC017 and ANAC013 TFs, acting as a negative regulator of their function. Moreover, the inactivation of RCD1 leads to increased expression of MDS genes regulated by both ANAC TFs, including AOXs, affecting the redox status of the chloroplasts. This leads to changes in chloroplasts ROS processing (including 2-Cys PRX) and to increased protection of the photosynthetic apparatus (Shapiguzov *et al*., 2019). These results support a role for RCD1 in integrating ROS signalling from both mitochondria and chloroplasts and as a modulator of nuclear gene expression. Another interaction point of the retrograde signals from both organelles, where ANAC17 is also involved, is the SAL1–PAP retrograde pathway. Target genes of ANAC017- and PAP-dependent signalling partially overlap. These results—the confirmation of SAL1 being dual targeted to chloroplasts and mitochondria and PAP accumulating in both organelles—suggest that SAL1 and PAP play an important role in MRS and not only in chloroplast retrograde signalling (Van Aken and Pogson, 2017). Additionally, the SAL1–PAP pathway converges with the RCD1-dependent pathway (Shapiguzov *et al*., 2019; Wang *et al*., 2020). The *rcd1* mutant has been seen to compromise the response to chloroplast ROS and also modify mitochondrial AOX respiration. In addition, an overlap in misregulated genes in the *rcd1* mutant with those affected by the PAP signalling pathway and genes of MDS, including AOX1a and the sulfotransferase SOT12 involved in generating PAP, has been reported. These effects are transduced in a retarded growth phenotype, and are more severe in the double *sal1/rcd1* mutants than in the single mutants (Shapiguzov *et al*., 2019). Interestingly, these authors have also shown that ROS (H_2_O_2_ and methyl viologen treatments) alter not only RCD1 abundance *in vivo*, but also its thiol redox state and oligomerization, which provides a feedback to fine-tune its activity (Fig. 2).

Another component involved in the regulation of *AOX1a* in response to MRR, as identified from genetic screening, is the Regulator of Alternative Oxidase1 (RAO1). RAO1 encodes the cyclin-dependent kinase E1 (CDKE1) that has been described as a central nuclear component integrating mitochondrial retrograde signals under various stress conditions, regulating a significant number of genes in the MRR regulon (reviewed by Crawford *et al.*, 2018), including *AOX1a* in response to H_2_O_2_ treatment and cold stress (Ng *et al*., 2013; Blanco *et al*., 2014). Also, CDKE1 can regulate the expression of light-harvesting complex B (*LHCB2.4*) and *AOX1a* in response to specific inhibitors of the photosynthesis electron transport chain (Blanco *et al*., 2014). Moreover, the *rao1* mutant alleles also demonstrate a GUN phenotype in response to redox changes in photosynthetic electron transport, implying that CDKE1 is a central nuclear component integrating mitochondrial and plastid retrograde signals. CDKE1 interacts in the nucleus with AKIN10 released from the ER, being one of the SnRK1 alpha subunit isoforms that acts as a metabolic master regulator in plants that is likely to be involved in the non-ROS signalling pathway, establishing a link between MRR and the overall energy signalling processes in plant cells (Ng *et al*., 2013; Wang *et al*., 2020). Interestingly, it has been shown that AKIN10 activity is strongly dependent on the redox status *in vitro* and that this redox sensitivity is conferred by a single Cys residue. However, the full extent of the described redox-modulated AKIN10 activity *in vivo* needs to be addressed (Wurzinger *et al*., 2017) (Fig. 2).

Besides transcriptional regulation, AOX is also post-translationally regulated by oxidation–reduction of the disulfide bridge formed between two conserved Cys residues (Martí *et al*., 2009; Nietzel *et al*., 2020). Mitochondrial TRX*o*1 reduces a wide number of mitochondrial proteins, including PRXIIF, GPXLs, some tricarboxylic acid (TCA) cycle enzymes and mtETC components, and AOX (Martí *et al*., 2009; Yoshida *et al*., 2013; Daloso *et al*., 2015). Compelling evidence indicates that AOX transcript and protein increase during salinity (Martí *et al*., 2011; Lázaro *et al*., 2013; Del-Saz *et al*., 2016), whereas a change in AOX isoform patterns was observed in knockout *Attrxo1* mutants under long-term salt stress (Sánchez-Guerrero *et al.*, 2019). Reduced *in vivo* AOX activity, but higher electron partitioning via AOX in the mutants also suggested that TRX*o*1 could help the reductive function of AOX to remain functional (Sánchez-Guerrero *et al*., 2019). In contrast to the effects caused by salinity, high light conditions did not reduce but increased the *in vivo* AOX activity in *Attrxo1* knockout mutants, while the AOX redox state was apparently unaltered, as observed under salinity (Florez-Sarasa *et al*., 2019). This suggests that other thiol redox systems such as GRXs might compensate for TRX*o*1 loss in the *Attrxo1* mutants, helping to prevent greater generation of ROS in mitochondria under the specific stress conditions studied, in this way participating in ROS-mediated retrograde signalling in these stress situations (Sánchez-Guerrero *et al*., 2021).

From all these data, there is no doubt concerning the role played by redox PTMs in chloroplast retrograde signalling. Because the main players of this process in the chloroplast also form part of the MRR, more studies are necessary in order to cast light on whether and how redox PTMs affect common or different proteins in mitochondria. The development of live imaging tools to monitor ROS and redox metabolism at a subcellular scale will be extremely helpful to better understand the signalling events that occur between subcellular compartments.

### Peroxisomes and apoplast in retrograde signalling

The involvement of H_2_O_2_ generated in peroxisomes in retrograde signalling has been described through the analysis of mutant plants in *CATALASE* (*CAT*) genes in *A. thaliana* and tobacco. These plants possess higher levels of peroxisomal H_2_O_2_, and show changes in transcriptional responses (Vandenabeele *et al*., 2003, 2004; Chaouch *et al*., 2010; Queval *et al*., 2012; Su *et al*., 2018; Terrón-Camero *et al*., 2022) that differ from those derived from the H_2_O_2_ produced in the chloroplasts (Sewelam *et al*., 2014). Peroxisomal H_2_O_2_ might participate in retrograde signalling through the OXI1/MPK (oxidative signal-inducible 1 kinase and mitogen-activated protein kinase) pathway (reviewed by Mielecki *et al*., 2020) (Fig. 2). In fact, OXI1, MPK11, and MPK13 were severely altered in a triple mutant *cat1cat2cat3* (Su *et al*., 2019) and, interestingly, OXI1 kinase activity was induced *in vitro* and *in vivo* by H_2_O_2_ (Rentel *et al*., 2004) although neither the underlying molecular mechanism nor the possible regulation by TRX has been described (Fig. 2).

ROS generation has been reported in the plasma membrane by RBOH (NADPH oxidase/respiratory burst oxidase homologue) enzymes producing O_2_·^–^ in the apoplast (Fig. 2). In this compartment, SOD dismutates O_2_·^–^ to H_2_O_2_, which can enter the cytoplasm through aquaporins (such as PIP1.4), where it modifies cytoplasmic proteins to regulate signalling. Stress situations such as drought or salinity are known to increase the phytohormone ABA that is sensed in the cytoplasm and nucleus by its receptors (PYR/PYL, Pyrabactin resistance 1/-like family) (Park *et al*., 2009). ABA induces RBOHs to generate ROS involved in the phytohormone signalling in Arabidopsis (Kwak *et al*., 2003). Related to this, we have recently described the redox regulation of PYR1 by TRX*o*1 in the nucleus of Arabidopsis and pea plants (De Brasi-Velasco *et al*., 2023), widening the role of TRXs to include the ABA-induced response under stress (Fig. 2). H_2_O_2_ is also known to trigger an influx of Ca^2+^ ions involved in signalling, and evidence of the role of a new H_2_O_2_ sensor in the process has recently been reported in Arabidopsis (Wu *et al*., 2020) (Fig. 2). Interestingly, this sensor is a plasma membrane protein, HPCA1, a leucine-rich-receptor kinase presenting four Cys residues in the extracellular domain activated by H_2_O_2_, which leads to autophosphorylation of the protein and activation of Ca^2+^ channels in guard cells, allowing integration of H_2_O_2_ and Ca^2+^ signalling. In this context, the presence of TRX has been reported in the plasma membrane and apoplast: AtTRX*h*9 has been associated with the plasma membrane and been seen to move from cell to cell (Meng *et al*., 2010), while OsTRX*h*1 is secreted into the extracellular space (Zhang *et al*., 2011). It would be interesting to deepen our knowledge of the redox regulation of HPCA1 and the possible role of TRXs in H_2_O_2_ sensing in the apoplast (Fig. 2).

## Redox regulation of gene expression mediated by TRX and GRX

Several ROS are generated in different cell compartments when exposed to abiotic stresses, H_2_O_2_ in particular being recognized as a signalling molecule. However, the way in which this signal is perceived and transmitted is still far from being understood, although a massive change in the transcriptome is evident. In fact, analysis of microarray datasets from plants grown under unfavourable conditions has revealed that transcripts responded specifically to the ROS species (Gadjev *et al*., 2006; Shaikhali and Wingsle, 2017). As an example, in different stress conditions, ^1^O_2_ induced the largest set of ROS-related genes while transcripts responsive to H_2_O_2_ and O_2_·^–^ were mainly repressed (Gadjev *et al*., 2006). The increased level of cellular ROS as a result of environmental stresses must enter the nucleus to modulate gene expression via nuclear sensors (He *et al*., 2018). Also, some ROS-responding elements in DNA have been described as inducing gene transcription under abiotic stress and ABA signalling (Allu *et al*., 2014) such as the G-box element (CACGTG) or the CORE element in the promoter of some antioxidant genes (Petrov and Van Breusegem, 2012), although this aspect is not the subject of the present review. Related to nuclear sensors, redox regulation usually induces conformation changes in key players of signalling events such as TFs or associated proteins, one interesting aspect being that this can occur in the cytosol and trigger nuclear translocation. In fact, redox-dependent conformational changes may expose the buried nuclear localization sequence, enabling nuclear import. As an example, the glycolytic enzyme glucose 6-phosphate dehydrogenase C (GAPDH-C) translocates under oxidative stress to the nucleus, where it functions as a transcriptional activator of glycolytic genes (Zhang *et al*., 2017). TFs are proteins that control gene expression through DNA binding, in this way promoting or suppressing transcription. Nuclear-encoded TFs are translocated into the cytoplasm where most of them are sequestered. Particularly under stress, they are translocated into the nucleus, the process being mediated by PTMs. Among them, oxidative PTMs are recognized as an important point of regulation through their action on TFs, with redoxins being key players in the direct interaction with them (Hopkins and Neumann, 2019). Related to this, the presence of thioredoxin (TRX*o*1) in the nucleus was reported by our group in pea leaves and TBY2 cells, where it is also localized in mitochondria, and some target proteins were identified in both organelles, as mentioned above (Martí *et al*., 2009; Calderón *et al*., 2017). Other components of the TRX system, such as NTR and TRX*h*, have also been identified in this organelle (reviewed by Martins *et al*., 2018), implying that redox regulation may be a key event for the nuclear function. Similarly, some mammalian TRXs accumulate in the nucleus under stress conditions (Wei *et al*., 2000), although pea TRX*o*1 was shown to be present in this cell compartment under non-stressed conditions, with a possible role protecting heterochromatin from oxidation, as proposed for mammalian PRDX5 (Kropotov *et al*., 2006).

**Fig. 3.**
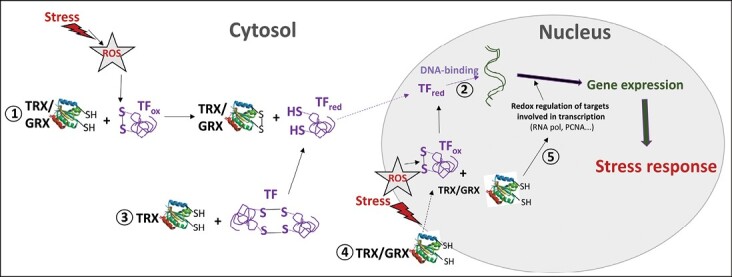
TRX/GRX-dependent redox regulation of gene transcription to induce a stress response. Under stress, generated reactive oxygen species (ROS) provoke oxidation of proteins involved in gene transcription. In this scenario, different possible mechanisms are triggered involving thioredoxin TRX or glutaredoxin GRX, such as: (1) reduction of oxidized (ox) TFs to their reduced (red) forms in the cytosol, thus facilitating nuclear translocation (dotted arrow); (2) reduction of oxidized TFs affecting DNA binding activity; (3) redox regulation of the oligomerization state of TFs; (4) stress-induced translocation of TRX/GRX to the nucleus, where they can (5) regulate protein targets involved in gene transcription (such as RNA polymerase, RNA pol; proliferating cellular nuclear antigen, PCNA). The different routes are derived from evidence reported in the literature, as described in the text.

In this review, considering mainly their cytosolic and nuclear localization, we summarize present knowledge of the role of TRXs in the regulation of redox sensors, especially their effect on stress-responsive TFs as essential targets involved in gene regulation. The DNA binding activity of TFs has been described as being redox regulated (D’Autreaux and Toledano, 2007), as has the involvement of sulfenic forms of Cys and disulfide bonds, as reported in yeast, microorganisms, and animals (reviewed by Antelman and Helmann, 2011; Sevilla *et al*., 2015; He *et al*., 2018). Some TFs are targets of TRX, for example GABP (GA-binding protein known as nuclear respiratory factor 2), which regulates the expression of nuclear-encoded mitochondrial proteins involved in oxidative phosphorylation (Martin *et al*., 1996). Another example is yeast TPX1, which required cytosolic TRX1 to be recycled from the oxidized form before translocation to the nucleus to activate PAP1 (Schippers *et al*., 2012; Calvo *et al*., 2013) (Fig. 3, point 1). Interestingly, some of these redox-sensitive TFs are known to induce several *TRX* genes and/or *NTR* gene expression, so they are also considered to be redox-controlled regulators (reviewed by Antelmann and Helmann, 2011).

Different TFs have been described as being specifically induced under a given stress, while others were frequently seen to be induced >5-fold in a variety of stress conditions (Gadjev *et al*., 2006). Among the last, two WRKY TFs, AtWRKY6 and AtWRKY75, together with a basic helix–loop–helix (bHLH) factor, were identified in most of the experiments. WRKY genes were shown to be induced under oxidative stress conditions and to regulate plant development and cell death (Ülker and Somiich, 2004; Guo and Gan, 2005). The W-box (TTGAC/T) is recognized by WRKY and, as an example, *A. thaliana TRXh5* expression has been associated with WRKY6 under oxidative stress (Laloi *et al*., 2004). Little is known about the possible redox regulation of WRKY TFs. Recently, it has been reported that WRKY25 is regulated by reducing and oxidizing conditions, which activate or repress its DNA binding activity, respectively (Fig. 3, point 2), although the biological effector is unknown and not all WRKYs showed this redox sensitivity: as examples, WRKY18 or WRKY53 seemed to be insensitive (Doll *et al*., 2020). Interestingly, dimerization of some of these TFs occurs as in the case of WRKY25, although little is known about its relationship with DNA binding activity. Other redox-sensitive TFs are the heat shock factors (HSFs), such as AtHSFA1a (Liu *et al*., 2013), in which the substitution of Cys residues by serine inhibited the expression of its target gene. The H_2_O_2_-dependent translocation of HSFA1D and HSFA8 is also dependent on specific Cys residues (Jung *et al*., 2013; Giesguth *et al*., 2015), so they could be possible candidates for TRX regulation. The maize R2R3-type MYB TFs also require reducing conditions for DNA binding, Cys49 and Cys53 being involved in the formation of a disulfide bond in oxidizing conditions (Heine *et al*., 2004), while the Cys-dependent redox regulation of basic region leucine zipper (bZIP) transcription factors has also been reported (Shaikhali *et al*., 2012). Another example is the HD-ZIP III class TFs involved in plant development, which present two conserved Cys residues, including Athb-9 which only showed DNA binding activity when these Cys were in the thiol state. Moreover, oxidized inactive Athb-9 was activated by *Escherichia coli* TRX (Comelli and Gonzalez, 2007).

Another mechanism that involves ROS and redox regulation of transcription is that of redox-dependent changes in the conformation state of target proteins, in relation to which TRX acts as an effector through structural changes on protein oligomerization (Fig. 3, point 3). For example, in plants, the homodomain of HD-Zip and glabra2 TFs is associated with a leucine zipper-like dimerization motif containing a set of conserved cysteines responsible for the dimerization and loss of activity under oxidizing conditions (Tron *et al*., 2002). Moreover, *E. coli* TRX, together with NADPH and NTR, were able to activate sunflower Hahb-10 and HAHR1 by reduction, provoking their monomerization. Another well-characterized example of redox regulation by TRX, TRX*h*5, has been reported as being involved in redox regulation of NPR1 through monomerization of cytosolic oligomers that induces its translocation to the nucleus to activate TGA TFs (Mou *et al*., 2003; Tada *et al*., 2008). Another example is sugarcane TRX*h*1, which was reported to regulate SsNAC23 that is involved in development and the response to cold stress (Ditt *et al*., 2011). Also the inhibition resulting from the oxidation of AtTCP15 and AtTCP16 TFs involved in cell proliferation was reverted by *E. coli* TRX and NTR (Viola *et al*., 2013), involving dimerization by oxidation reverted by the redox system. It has recently been described that under cold stress, Trx*h*2 translocates to the nucleus, where it binds to C-repeat-binding factors (CBFs), allowing monomerization of inactive oxidized S–S bridges and the reduction of monomers (active) (Lee *et al*., 2021) (Fig. 3, point 4). The monomers then bind to the C-repeat (CRT)/dehydration-responsive element (DRE) sequence in the promoters of target genes to induce their expression and the cold stress response. Another redox sensor is the plant-specific DNA-binding WHIRLY, which, in control conditions, formed oligomers in the chloroplasts, while alteration of the redox state of the photosynthetic complex under stress has been proposed to induce its monomerization and translocation to the nucleus (Foyer *et al*., 2014).

Related to GRXs, Zander *et al*. (2012) showed that at least 17 of the 21 CC-type GRX (ROXYs) encoded by the *A. thaliana* genome interact with bZIP TF TGA2 in a yeast two-hybrid system. GRXs may play an important role in hormone biology, probably by regulating the target proteins involved in hormone signalling pathways such as several TFs. In *A. thaliana*, ROXY19/GRX480 has been shown to suppress the activation of the ORA59 (OCTADECANOID-RESPONSIVE ARABIDOPSIS AP2/ERF-domain protein 59) promoter by the TF EIN3 (ETHYLENE INSENSITVE 3) through TGA2, TGA5, and/or TGA6. In this way, ROXY19/GRX480 negatively regulates the expression of jasmonic acid (JA)/ethylene (ET)-induced defence genes (Zander *et al*., 2012). Also, after salicylic acid (SA) treatment, critical Cys residues of NPR1 and TGA1 are in their reducing state (Despres *et al*., 2003), and AtGRX480, which is induced by SA and requires NPR1, was seen to interact with the TGA2.2 TF in a yeast protein interaction screening. Moreover, this GRX is a negative effector of the JA-responsive gene *PDF1.2*, representing a potential regulator of SA/JA antagonism (Ndamukong *et al*., 2007).

The involvement of GRX redox proteins as TGA-interacting factors in conditions that alter the redox status of the cell, such as development and environmental constraints, has also been demonstrated, indicating that they may be biologically relevant in these situations. Regarding abiotic stress, AtGRXS8 interacts with TGA1 and TGA4 TFs, which are central regulators of early transcriptional responses to nitrate in *A. thaliana* roots, acting as a negative regulator of the primary transcriptional response to nitrate availability (Patterson *et al*., 2016; Ehrary *et al*., 2020; Ota *et al*., 2020). Similarly to *A. thaliana*, a cassava CC-type GRX, MeGRXC3, regulates sensitivity to mannitol-induced osmotic stress tolerance by interaction with TGA2 and TGA5 in the nucleus, while positively regulating several stress-related TFs including ERF6 and ORA59 (Ruan *et al*., 2022). As another example, MeGRXC15 may regulate drought responses by interacting with MeTGA074 (Ruan *et al*., 2018) and, in tomato, TGA2 is activated via GRXS25-dependent post-translational redox modification to mediate in brassinosteroid (BR)-induced pesticide metabolism (Hou *et al*., 2019). Within the *A. thaliana* nuclear factor NF-Y family that participates in plant development and stress responses, Knuesting *et al*. (2015) have shown that NF-YC11/NC2α interacts with GRXS17 and that they act together to control plant development in relation to environmental conditions. The authors speculated that GRXS17 may control the redox state of NF-YC11/NC2α and thus regulate its function.

Related to development, it has been demonstrated that AtROXY8 and AtROXY9 affect hyponastic growth in *A. thaliana*, probably by regulation of TGA1 and TGA4 (Li *et al*., 2019). However, although TGA1 and TGA4 also interact with ROXYs 18 and 19/GRX480, they do not interfere with hyponastic growth, suggesting that, despite promiscuous TGA–ROXY interactions, there might be functional specificities of individual ROXYs for distinct TGAs affecting different mechanisms (Li *et al*., 2019). The land plant-specific GRXs ROXY1 and ROXY2 that are involved in microspore structure, anther development, and petal number in *A. thaliana* have been shown to interact with TGA TFs such as TGA2, TGA3, TGA8 (PERIANTHIA, PAN), TAG9, and TGA10 (Xing *et al*., 2005; Murmu *et al*., 2010; Li *et al*., 2011; Gatz, 2013). Using mutated variants of ROXY in some of the Cys residues, the importance of some of these residues in the redox regulation of PAN has been established, although the mechanism by which 2-Cys (CC-type) GRXs regulates TGA motifs is unknown (Li *et al*., 2011). More recently, the interaction in the nucleus of ROXY1/2 with PAN to regulate petal development has been shown to occur under reducing and not under oxidizing conditions (Fig. 3, point 4), ROXY1 being co-localized with the active form of RNA polymerase II (Gutsche *et al*., 2017). This suggests the existence of another point of control of the gene expression through redox regulation of protein targets involved in that process (Fig. 3, point 5). Related to this, proliferating cell nuclear antigen (PCNA), a key component of the DNA replication and repair machinery (Strzalka and Ziemienowicz, 2011) is regulated by PsTRX*o*1 during DNA repair, division, and proliferation of tobacco BY-2 cells (Calderón *et al*., 2017). PCNA is involved in the development process, including the transition of buds from a quiescent to an actively growing state, which depends on the resumption of cell division and elongation (Porcher *et al*., 2020), while TRX and GRX in bacteria, yeast, and mammals have been reported to act as electron donors for ribonucleotide reductase (RNR) during DNA synthesis (reviewed by Meyer *et al*., 2012; Sengupta and Holmgren, 2014). Another component involved in modulation of RNA polymerase II is the Mediator (MED) multiprotein complex, a well conserved transcriptional co-activator that acts as a bridge between TFs and the polymerase. CDK1 is one of the components of the MED complex and it has been suggested that signals from cell compartments are perceived by the CDK1–KIN10 complex and transmitted to MED, which transcribes the instructions to the polymerase II machinery to initiate transcription as part of the retrograde signalling pathway in *A. thaliana* (Ng *et al*., 2013; Shaikhali *et al*., 2015). Interestingly, three Cys-containing MED subunits (MED10a, MED28, and MED32) form oligomers by means of intermolecular disulfide bonds that are reduced *in vitro* by the TRX and GSH–GRX systems, with the DNA binding activity of MED being affected by the redox state (Shaikhali *et al*., 2015, 2016).

All the above-mentioned interactions reinforce the importance of redox-sensitive transcriptional regulation in the adaptation of plants to different scenarios to cope with the imposed stress, pointing to redoxins as key elements that allow the rapid and specific on/off switching of gene expression. Hence, redox regulation of nuclear function is emerging as an important issue in plant acclimation, while an interesting challenge for research in redox biology will be the search for and identification of functional evidence of the ROS sensors and redox switches. Proteome approaches have identified several nuclear proteins prone to redox modifications, suggesting that redox regulation, TRXs, and GRXs may be regarded as good candidates to exert this function.

## Conclusion

Redox regulation is emerging as a key control point in plant metabolism, and has been seen to involve several players as sensors, transducers, and responders. TRXs and GRXs are considered as transducers during redox signalling and, in this review, we have analysed their action on specific target proteins with a special focus on organelle retrograde communication and on gene transcription during stress response. Modulation of the PTMs responsible for changes in the redox state of thiols in proteins will help the plant cellular metabolism to cope with stress situations, allowing adaptation. Redox regulation of such important processes as organelle communication with the nucleus and modulation of gene transcription has an impact on the control of cell growth and development in plants, with TRXs and GRXs also playing a role This regulation represents only part of the complex process of response, not only during normal growth situations but also, importantly, to the increasingly unfavourable conditions for plant production. Many gaps remain in our knowledge of the signalling pathways described, and several players still need to be identified in our attempts to improve plant performance not only in models such as Arabidopsis but also in crop plants. The development of new techniques in genome editing, ‘omic’ approaches, and the detection of PTMs will help us to understand the role of redox signalling in developmental and stress responses.
